# Retroperitoneal Basidiobolomycosis Mimicking a Malignant Neoplasm

**DOI:** 10.14740/gr2138

**Published:** 2026-06-16

**Authors:** Abdullah Saad Alqahtani, Mahmoud Rezk Abdelwahed Hussein, Mohammed Abdulrahman Alshehri, Saeed Ali Alqahtani, Turki Mohammad Hamdi, Ali Amer Al Shehri, Mohammed Kamal Semary, Asim A. Elyas, Toka Mahmoud Rezk A. Hussein

**Affiliations:** a Department of Surgery, Armed Forces Hospital Southern Region, Khamis Mushait, KSA; b Pathology Department, Assiut University Hospitals, Assiut, Egypt; c Department of Radiology and Medical Imaging, Armed Forces Hospital Southern Region, Khamis Mushait, KSA; d Department of Internal Medicine, Armed Forces Hospital Southern Region, Khamis Mushait, KSA; e Faculty of Medicine, Sohag University, Sohag, Egypt

**Keywords:** Liver, Basidiobolomycosis, Immunocompetent, *Basidiobolus ranarum*

## Abstract

Basidiobolomycosis is a rare and potentially fatal fungal infection caused by *Basidiobolus ranarum* (*B. ranarum*). Retroperitoneal or mesenteric basidiobolomycosis can mimic malignant tumors clinically and radiologically. We describe a 15-year-old girl who developed retroperitoneal and mesenteric fungal masses due to *B. ranarum*. The patient presented with gradual onset of jaundice, significant weight loss, abdominal pain, and vomiting. Computed tomography revealed masses in the retroperitoneal and mesenteric regions with radiological findings suggestive of malignant neoplasm. Tru-Cut biopsy of the retroperitoneal mass confirmed basidiobolomycosis on histological examination. Following diagnosis, antifungal therapy was initiated with intravenous voriconazole (4 mg/kg/day for 2 weeks), followed by oral itraconazole (200 mg every 12 h). Regular follow-up through May 2026 demonstrated significant clinical improvement without major adverse effects. This case underscores the importance of recognizing basidiobolomycosis as a potential cause of retroperitoneal or mesenteric masses that may be misinterpreted as malignant tumors. Non-specific symptoms often delay diagnosis and appropriate treatment. Clinicians in regions where *B. ranarum* is endemic should include basidiobolomycosis in the differential diagnosis when evaluating patients with retroperitoneal or mesenteric masses.

## Introduction

### An overview of basidiobolomycosis fungal infection

Basidiobolomycosis is a potentially fatal fungal disease attributed to *Basidiobolus ranarum* (*B. ranarum*), which belongs to the Zygomycetes family. This condition can manifest in two distinct forms depending on the host’s immune status. The Entomophthorales order affects immunocompetent individuals, whereas the Mucorales order is associated with immunocompromised hosts [1]. The initial identification of *B. ranarum* dates back to 1886, when Eidam discovered it in the feces of frogs. The fungus was later isolated from decomposing plant material in Arizona in 1955 [2, 3]. Notably, the genome of *B. ranarum* is approximately 10 times larger than that of typical fungal pathogens, enabling it to retain overlapping functional genes [4].

Basidiobolomycosis is predominantly observed in tropical and subtropical regions, including Arizona in the United States, Saudi Arabia, Iran, Oman, and India [1, 5]. In the Jazan region of Saudi Arabia, geckos serve as carriers of *B. ranarum*, posing a significant risk for the development of basidiobolomycosis, particularly in children [5]. The organism is widely present in decaying vegetation, fruits, soil, and the intestines of various animals, including bats, reptiles, fish, and amphibians [6].

**Figure 1:**
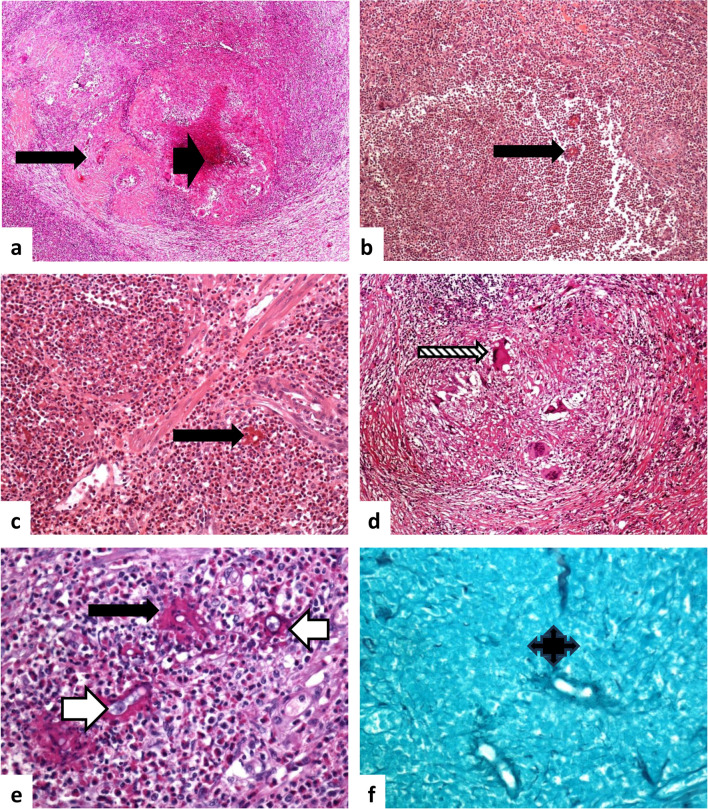
Histological features of basidiobolomycosis. (a) Basidiobolomycosis involving the pericolic tissue and muscular layer of the colon, with extensive eosinophil-rich granulomatous inflammation. Multiple fungal hyphae are surrounded by sleeves of eosinophilic material (a: × 20, b: × 40, c: × 100, e: × 400; Splendore–Hoeppli phenomenon, long black arrow), along with areas of necrosis (a: short black arrow). Foreign-body giant cells with surrounding inflammatory reaction are present (d: ×200, patterned long arrow). The fungal elements display features similar to amoebic trophozoites, characterized by foamy cytoplasm and a prominent nucleolus without heterochromatin (e: white long arrow). (f) Gomori methenamine silver (GMS) staining highlights fungal hyphae, enhancing visualization of the fungal cell walls (quad arrow; × 400). Source of figure: Prof Dr. Hussein.

The microbiological culture is rarely used for the diagnosis of basidiobolomycosis. In culture, the fungal colonies of *B. ranarum* exhibit a distinctive waxy or glabrous appearance with radial folds covered by aerial hyphae and are characterized by a yellow-grayish coloration [6]. The diagnosis of gastrointestinal basidiobolomycosis typically relies on histological examination of the lesional tissue, which reveals fungal hyphae encased in sleeves composed of thick, hyalinized eosinophilic material—a phenomenon termed the “Splendore–Hoeppli phenomenon.” This phenomenon represents an immunological response that forms a protective barrier around the fungal elements, thereby facilitating the persistence and chronic nature of *B. ranarum* infection [7] (Fig. 1). The average diameter of the hyphae is approximately 9 µm, with individual diameters ranging from 5.0 to 20 µm. The lesional tissues in basidiobolomycosis demonstrate granulomatous inflammation with dense infiltration by eosinophils, which are specialized white blood cells that play a central role in the innate immune response against fungal pathogens including *B. ranarum* infection [7]. These cells express receptors for various adhesion molecules, cytokines, and chemokines critical for mediating inflammatory responses. As components of the innate immune system, eosinophils employ pattern recognition receptors—including Toll-like receptors and C-type lectin receptors—to detect microbial components known as pathogen-associated molecular patterns (PAMPs) and signals from damaged or dying cells referred to as danger-associated molecular patterns (DAMPs). Recognition of PAMPs or DAMPs activates leukocyte effector mechanisms, thereby initiating innate immune and inflammatory responses [8–10].

Delayed diagnosis of basidiobolomycosis is often associated with poor prognosis and increased mortality rates, estimated at approximately 20%. Its management typically involves surgical excision combined with antifungal therapy [11–13].

### Gastrointestinal basidiobolomycosis

Basidiobolomycosis is a rare and aggressive fungal infection that only infrequently involves the gastrointestinal system. Its rarity may be attributed to the absence of multiple pathogenic strains of the causative fungus and the development of strong acquired immunity following prior subclinical infections in endemic regions [4, 12]. The first documented case of gastrointestinal basidiobolomycosis was reported in 1964 in Nigeria during the autopsy of a 6-year-old child. The infection may result from the ingestion of food contaminated with *B. ranarum* [14].

Basidiobolomycosis typically presents as a subcutaneous infection but can occasionally involve the gastrointestinal tract. The colon—particularly the right side—is the most commonly affected site, followed by the small intestine, liver, and gallbladder [1, 11]. This condition is frequently misdiagnosed as a neoplastic or inflammatory abdominal condition and predominantly affects immunocompetent children, especially boys, who often have no prior history of infectious disease exposure [5, 11, 13, 15].

Several case reports and case series have documented instances of abdominal basidiobolomycosis [1, 12, 16]. Pezzani et al analyzed the clinical features of 102 cases of basidiobolomycosis and identified abdominal pain as the most common presenting symptom, followed by weight loss, abdominal distension, vomiting, diarrhea, and fever. Abdominal masses were reported in 30.4% of cases [12]. Common laboratory findings included elevated erythrocyte sedimentation rate, increased C-reactive protein (CRP) levels, eosinophilia, and neutrophilic leukocytosis. Surgery combined with antifungal therapy was the predominant treatment modality, used in 77.5% of patients. An unfavorable outcome was observed in 18.6% of cases overall [12].

In Saudi Arabia, Al Haq et al reported a large case series of 12 pediatric patients with gastrointestinal basidiobolomycosis (aged 16 months to 8 years, diagnosed between January 2012 and December 2019), including two patients with retroperitoneal involvement [13]. Patient 2 was a 4-year-old male from Jeddah with an 8-week history of fever, abdominal pain, a palpable mass, weight loss, and diarrhea. Laboratory findings showed leukocytosis, anemia, marked eosinophilia, and elevated CRP. Computed tomography (CT) revealed a large retroperitoneal mass with portal vein thrombosis. Culture was negative, and frozen section showed only inflammatory changes. He required multiple laparotomies and bowel resection, ultimately developing short bowel syndrome [13]. Patient 9 was a 6-year-old male from the Al Ardiyat area who presented after 4 weeks of fever, abdominal pain, and weight loss. Laboratory findings showed leukocytosis, mild anemia, marked eosinophilia, and elevated CRP. CT revealed a large right-sided retroperitoneal mass. Culture was negative, and he underwent laparotomy with biopsy [13].

Here, we present a case of basidiobolomycosis-induced inflammatory fungal lesions that were initially misdiagnosed as neoplasms in the retroperitoneal and mesenteric regions. This case report, along with a comprehensive review of the literature, examines the diagnostic challenges associated with this condition and emphasizes the importance of distinguishing it from malignant tumors.

## Case Report

### History and clinical presentation

In October 2024, a 15-year-old female patient from Khamis Mushait City in the Aseer region of the Kingdom of Saudi Arabia presented with a 3-year history of non-specific symptoms, including intermittent right upper quadrant abdominal pain, vomiting, weight loss, anorexia, and night sweats. She subsequently experienced worsening abdominal pain and jaundice, prompting referral to the surgical clinic for further evaluation. On physical examination, significant findings included weight loss and vague abdominal pain.

### Laboratory findings

Laboratory investigations revealed multiple abnormalities, as summarized in Table 1. Notably, serum calcium was low at 2.19 mmol/L (reference range: 2.23–2.58 mmol/L), phosphorus at 0.81 mmol/L (reference range: 1.07–2 mmol/L), and sodium at 133.00 mmol/L (reference range: 133–143 mmol/L). Elevated serum values included potassium at 5.50 mmol/L (reference range: 3.5–5.1 mmol/L), alkaline phosphatase at 381.00 U/L (reference range: 67–372 U/L), gamma-glutamyl transpeptidase (GGT) at 270.00 U/L (reference range: 8–23 U/L), total protein at 87.00 g/L (reference range: 61–80 g/L), direct bilirubin at 58.90 µmol/L (reference range: 1.7–8.6 µmol/L), alanine aminotransferase at 20.00 U/L (reference range: 8–29 U/L), and aspartate aminotransferase at 21.00 U/L (reference range: 14–37 U/L).

**Table 1:** Laboratory Findings of the Patient’s Investigations

Description	Result	Unit	Reference range
Amylase - serum	68.00	U/L	28–100
Bone profile			
Calcium - serum	2.19 (L)	mmol/L	2.23–2.58
Albumin modular	35.00	g/L	35–48
Magnesium - serum	0.79	mmol/L	0.66–1.07
Phosphorus - serum	0.81 (L)	mmol/L	1.07–2
Alkaline phosphatase	381.00 (H)	U/L	67–372
Cardiac enzymes			
Aspartate aminotransferase	21.00	U/L	14–37
Creatine kinase	35.00	U/L	28–170
Lactic dehydrogenase	195.00	U/L	122–234
Complete blood count with differential			
White blood cell count	12.70	10^9^/L	
Red blood cell count	4.62	10^12^/L	
Hemoglobin	8.95	g/dL	
Hematocrit	30.10	%	
Mean corpuscular volume	65.10 (L)	fL	76–96
Mean corpuscular hemoglobin	19.40	pg	
Mean corpuscular hemoglobin concentration	29.70 (L)	g/dL	32–36
Red cell distribution width	30.50 (H)	%	11–14
Platelet count	1,010	10^9^/L	
Mean platelet volume	9.27	fL	
Neutrophil percentage	81.90	%	
Lymphocyte percentage	6.82	%	
Monocyte percentage	10.60	%	
Eosinophil percentage	0.04	%	
Basophil percentage	0.08	%	
Immature granulocyte percentage	0.60	%	
Neutrophil absolute count	10.4 (H)		2–7.5
Lymphocyte absolute count	0.86		
Monocyte absolute count	1.34		
Eosinophil absolute count	0		
Basophil absolute count	0.01		
Immature granulocyte absolute count	0.08		
Nucleated red blood cell	0.00	%	
Creatine kinase-MB	0.50	ng/mL	0.46–2.04
C-reactive protein	149.90	mg/L	< 5
hs-troponin-I	< 5.1	pg/mL	8.4–18.3
Liver function tests			
Total protein modular - serum	87.00 (H)	g/L	61–80
Total bilirubin	78.40	µmol/L	< 34.2
Albumin modular	35.00	g/L	31–48
Direct bilirubin	58.90 (H)	µmol/L	1.7–8.6
Alanine aminotransferase	20.00	U/L	8–29
Aspartate aminotransferase	21.00	U/L	14–37
Alkaline phosphatase	381.00 (H)	U/L	67–372
Gamma-glutamyl transpeptidase	270.00 (H)	U/L	8–23
Renal profile			
Sodium - serum	133.00	mmol/L	133–143
Potassium - serum	5.50 H	mmol/L	3.5–5.1
Chloride - serum	103.00	mmol/L	98–115
Creatinine - serum	36.90	µmol/L	27–88
Blood urea nitrogen - serum	4.40	mmol/L	2.5–7.85

Hematological evaluation revealed low mean corpuscular volume (MCV) at 65.10 fL (reference range: 76–96 fL) and low mean corpuscular hemoglobin concentration (MCHC) at 29.70 g/dL (reference range: 32–36 g/dL), while red cell distribution width (RDW) was elevated at 30.50% (reference range: 11–14%) and the absolute neutrophil count (NEUT ABS) was increased at 10.4 × 10^3^/µL (reference range: 2–7.5 × 10^3^/µL). A complete summary of the patient’s laboratory results is presented in Table 1.

### Radiological findings

**Figure 2:**
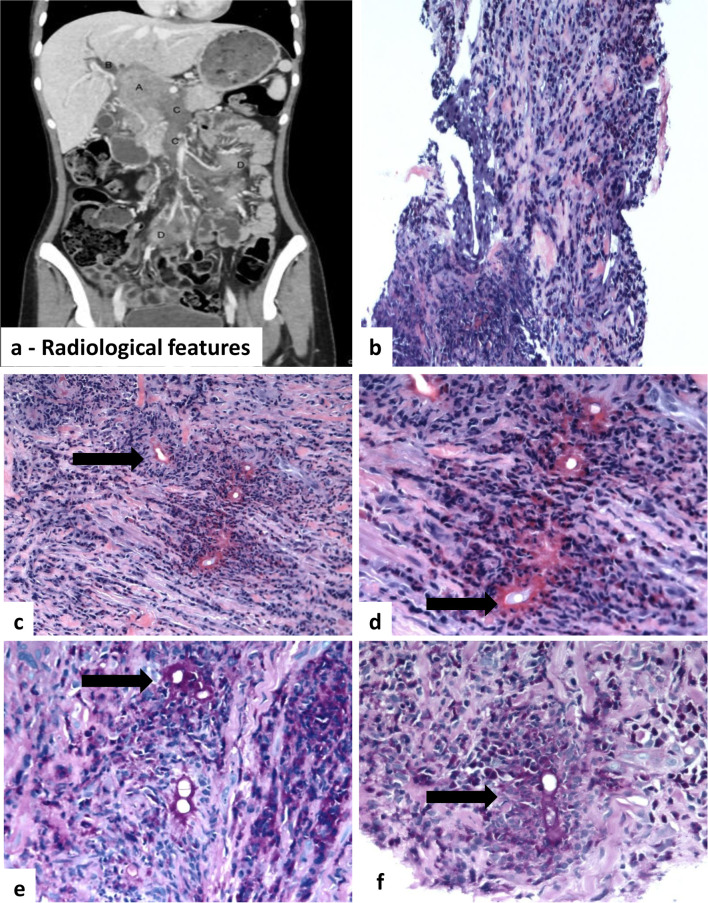
Radiological and histological features of a basidiobolomycosis-induced inflammatory retroperitoneal fungal mass. (a) Radiological features: porta hepatis mass with biliary and vascular involvement. Contrast-enhanced CT images demonstrate a 6.5 × 4.3 cm enhancing mass in the porta hepatis (A, black arrow). The mass causes severe narrowing of the common bile duct (B, white arrowhead), resulting in marked intrahepatic biliary dilatation. There is invasion of the portal vein and superior mesenteric vein (C, white arrow). Additional metastatic deposits are seen in the small bowel mesentery (D, curved arrow). (b) Histological sections demonstrate patchy and vaguely nodular aggregates of inflammatory cells infiltrating the connective tissue. (c)–(e) Fungal hyphae surrounded by eosinophil-rich mixed inflammatory cell infiltrates. Multiple thin-walled, non-septate hyphae (thin arrow) are encased by brightly hyaline eosinophilic material (Splendore–Hoeppli phenomenon, thick arrow). Areas of necrosis are present within the fibroconnective tissue. (f) Fungal hyphae highlighted by periodic acid–Schiff (PAS) stain. Magnifications: a: × 20; b: × 100; c–e: × 400, H&E; f: × 400, PAS).

CT scans revealed a hypo-enhancing soft tissue mass in the paravertebral retroperitoneal region, extending from the pelvic area into the upper abdomen. Additionally, a lobulated soft tissue mass measuring 50 × 43 mm was identified at the porta hepatis, closely associated with the pancreatic head and the second portion of the duodenum. This lesion demonstrated peripheral infiltration and invasion of the portal vein, causing compression of the bile ducts and resulting in upstream dilation of both the common bile duct and intrahepatic ducts (Fig. 2). The portal vein appeared narrowed, with multiple collateral vessels indicative of portal vein occlusion. The gallbladder was contracted, with a dilated common bile duct and multiple collateral vessels in the porta hepatis region. The liver maintained a smooth contour without focal lesions, while an ill-defined soft tissue mass was observed along the mesenteric vessels, associated with mild ascites. Mild enlargement of mediastinal lymph nodes and a small pericardial effusion were also noted.

Radiological assessment suggested the possibility of metastasis or lymphoma, prompting a Tru-Cut needle biopsy of the porta hepatis mass. The patient subsequently underwent endoscopic retrograde cholangiopancreatography (ERCP), followed by percutaneous transhepatic cholangiography (PTC) with external drainage to relieve jaundice. A summary of the radiological findings is shown in Figure 2.

### Histopathological findings

**Figure 3:**
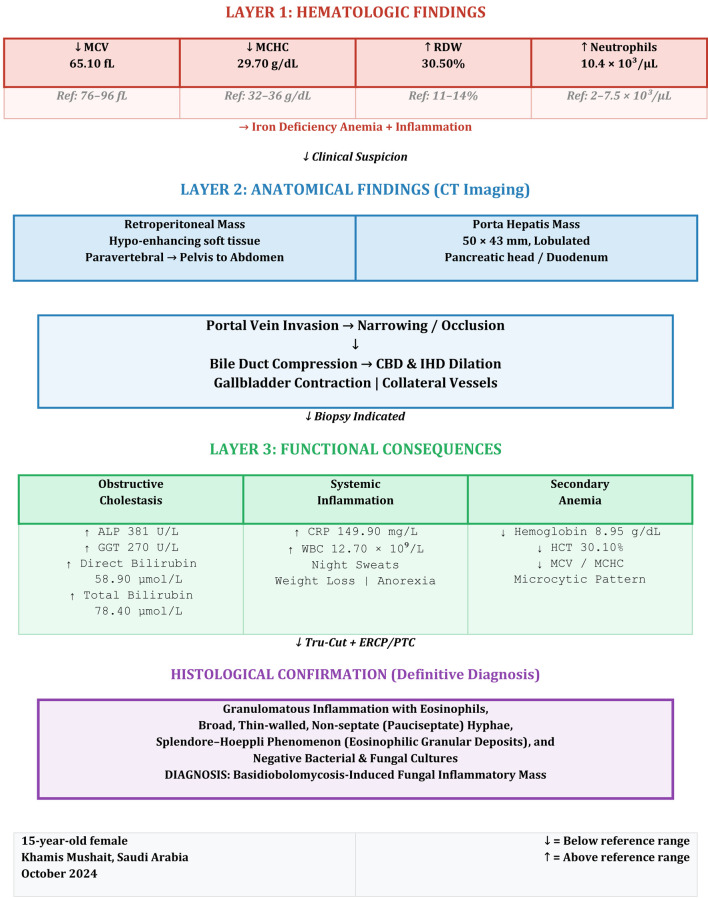
Three-layered diagnostic framework in retroperitoneal basidiobolomycosis. Layer 1 (hematologic): iron deficiency anemia (↓MCV 65.10 fL, ↓MCHC 29.70 g/dL, ↑RDW 30.50%) with inflammation (↑neutrophils 10.4 × 10^3^/µL). Layer 2 (anatomical): CT revealed a retroperitoneal soft tissue mass and a 50 × 43 mm lobulated porta hepatis mass causing portal vein invasion, bile duct compression (CBD and IHD dilation), and gallbladder contraction. Layer 3 (functional): obstructive cholestasis (↑ALP 381 U/L, ↑GGT 270 U/L, ↑direct bilirubin 58.90 µmol/L), systemic inflammation (↑CRP 149.90 mg/L, ↑WBC 12.70 × 10^9^/L), and secondary anemia (↓hemoglobin 8.95 g/dL). Histological confirmation: Tru-Cut biopsy demonstrated granulomatous inflammation with eosinophils, broad pauciseptate hyphae, and Splendore–Hoeppli phenomenon, establishing the diagnosis of basidiobolomycosis despite negative cultures. ALP: alkaline phosphatase; GGT: gamma-glutamyl transferase; CBD: common bile duct; IHD: intrahepatic ducts; MCV: mean corpuscular volume; MCHC: mean corpuscular hemoglobin concentration; RDW: red cell distribution width; CRP: C-reactive protein; WBC: white blood cell count.

Histopathological examination revealed granulomatous inflammation with a prominent infiltrate of eosinophils, along with lymphocytes, epithelioid cells, histiocytes, and occasional foreign-body giant cells. This inflammatory response was observed in proximity to a limited number of fungal elements, with minimal residual liver parenchyma. The fungal structures consisted of broad, thin-walled, non-septate hyphae surrounded by amorphous eosinophilic granular deposits, identified as Splendore–Hoeppli bodies. Both bacterial and fungal cultures were negative. The diagnosis of basidiobolomycosis-induced inflammatory fungal masses was therefore established solely based on histological findings, as illustrated in Figure 1. Here we propose a practical three-layered diagnostic framework for the diagnosis of abdominal basidiobolomycosis. A schematic representation of this framework is depicted in Figure 3.

### Treatment and follow-up

Following the diagnosis, a multidisciplinary discussion involving the patient’s family was conducted, and the decision was made to initiate antifungal therapy. Intravenous voriconazole was administered at a weight-based dose of 4 mg/kg/day for 2 weeks, followed by oral itraconazole 200 mg every 12 h. Regular follow-up until May 2026 to assess therapeutic response and potential side effects demonstrated significant clinical improvement, with no major adverse effects observed.

## Discussion

This study presents an unusual case of basidiobolomycosis characterized by obstructive jaundice and an infiltrative process, specifically presenting as multiple retroperitoneal and mesenteric masses. The clinical manifestations, including fever, vomiting, abdominal pain, and the presence of multiple retroperitoneal and mesenteric masses, align with findings documented in earlier studies [1, 5, 11, 13]. The precise mechanism by which *B. ranarum* induces fungal mass formation in the retroperitoneal and mesenteric tissues remains largely unclear. It is hypothesized that the patient acquired the infection through consumption of food or water contaminated with this fungus, likely originating from soil or plants [10]. Subsequent spread of fungal elements into the surrounding retroperitoneal and mesenteric tissues triggered an inflammatory response characterized by recruitment of inflammatory cells, granulomatous reaction, and formation of exuberant granulation tissue.

In this case, consistent with previous reports [5, 12, 13], the basidiobolomycosis-induced inflammatory fungal masses were initially misdiagnosed as malignant lymphoma. Histological examination of tissue samples confirmed the diagnosis of basidiobolomycosis. The patient received antifungal therapy for 18 months, resulting in significant clinical improvement, with no residual mass lesions noted at the 19-month follow-up.

### Eosinophilia in basidiobolomycosis

Although eosinophilia has been frequently reported in previous cases of basidiobolomycosis [1, 5, 11, 13], the patient in this case did not show peripheral blood eosinophilia, with an eosinophil percentage of 0.04% and an absolute eosinophil count of 0 × 10^9^/L (both within normal limits). This finding contrasts with the characteristic eosinophilia described in the literature, where elevated levels of blood and tissue eosinophils are commonly reported [1, 5, 11, 13]. The absence of peripheral eosinophilia in our patient may be attributed to the retroperitoneal localization of the infection, the early stage of disease at presentation, or individual immunological variability. In contrast, histopathological examination of the excised tissue revealed eosinophilic infiltration, consistent with the granulomatous inflammatory response typically associated with fungal infection including *B. ranarum* infection. Based on these observations, we propose that in pediatric patients presenting with a retroperitoneal mass or gastrointestinal symptoms, the presence of tissue eosinophilia—particularly in the absence of allergies or autoimmune disorders—should raise consideration of a potential underlying fungal infection, including basidiobolomycosis. Moreover, physicians should be vigilant to the fact that the absence of peripheral blood eosinophilia does not exclude this diagnosis, and tissue biopsy remains essential for definitive confirmation [1, 5, 11, 13].

### Diagnosis of basidiobolomycosis

Diagnosis of basidiobolomycosis is typically based on histological examination rather than culture. Consistent with previous studies [1, 5, 11], the diagnosis in the present case was established through distinctive histopathological features rather than relying on culture results. Although culture on Sabouraud agar at 20–30 °C for 2–3 days is considered the gold standard for diagnosing basidiobolomycosis, its practical utility is limited due to potential delays in initiating treatment. The culture process is often inefficient, with poor yield, and may take up to 4 weeks to produce results. In contrast, timely diagnosis can be achieved through tissue biopsy, which demonstrates characteristic histological features and can significantly reduce both morbidity and mortality associated with basidiobolomycosis [11, 13, 15].

Detection of *B. ranarum* infection can be performed using immunodiffusion; however, the sensitivity of this method remains debated. Several studies have highlighted the utility of molecular techniques, particularly polymerase chain reaction (PCR), for diagnosing basidiobolomycosis. Optimal results are achieved when DNA is extracted from formalin-fixed, paraffin-embedded lesional tissues. Despite its effectiveness, this approach is not widely implemented in many healthcare facilities, primarily due to the rarity of basidiobolomycosis cases [17–20].

### Antifungal therapy versus surgical intervention in basidiobolomycosis-induced fungal masses

In the present case, antifungal therapy was chosen to manage the basidiobolomycosis-associated masses rather than surgical intervention, in alignment with findings from previous studies [5, 13, 16, 21]. Evidence indicates that surgical excision of these fungal masses may not significantly alter disease progression or improve outcomes [12, 13]. Moreover, surgery can increase patient morbidity by damaging the bowel and adjacent structures, potentially leading to complications such as fistula formation. Additional risks associated with surgical procedures include disseminated fungal infection, sepsis, septicemia, wound dehiscence, and anaphylactic shock [5, 12, 13]. Abd El Maksoud et al reported the outcomes of surgical management in 22 patients with colonic basidiobolomycosis at Aseer Central Hospital in the Kingdom of Saudi Arabia. All patients underwent colonic resection followed by antifungal therapy with voriconazole. Unfortunately, three patients died within 6 months post-surgery due to disease progression, and four patients developed severe wound infections [21].

### Defense mechanisms in zygomycotic infections

Various host defense mechanisms are employed to counteract zygomycotic infections, including the sequestration of iron molecules, the production of reactive oxygen radicals, and the release of metabolites that target and disrupt fungal structures. Additionally, polymorphonuclear leukocytes and histiocytes play a critical role in eliminating fungal organisms by secreting defensins, which are potent cationic peptides. The progression of zygomycosis is closely associated with the host’s ability to regulate and limit iron availability within lesional tissues, as iron is essential for fungal growth. Furthermore, fungal elements can invade host blood vessels, thereby facilitating vascular dissemination and the spread of infection throughout the organism [1, 7, 22].

### Conclusions and key lessons from our study

In conclusion, the insights from our current and previous investigations [1, 7, 8], along with findings from prior studies [11, 13], highlight several important points. In pediatric patients, the presence of non-specific abdominal symptoms—such as pain, fever, loss of appetite, and vomiting—combined with laboratory findings of anemia, neutrophilic leukocytosis, or eosinophilia, and the detection of an abdominal mass on radiological imaging, should raise a high index of suspicion for basidiobolomycosis. The clinical and radiological manifestations of basidiobolomycosis are largely non-specific and often mimic inflammatory conditions (such as inflammatory bowel disease and diverticulitis), infectious diseases (including amebiasis, abscesses, and tuberculosis), or neoplastic processes (such as lymphoma, sarcoma, or carcinoma) [1, 7, 22]. Diagnosing basidiobolomycosis is therefore challenging and requires a high degree of vigilance from clinicians, radiologists, and pathologists. Additionally, it is crucial to raise public awareness about basidiobolomycosis, particularly in regions where the disease is endemic [1, 7, 12, 13, 22].

### Strengths and limitations of the study

The three-layered approach is a notable strength of our study, providing a structured, clinically oriented framework that integrates hematologic, anatomical, and functional data. This approach may facilitate earlier recognition of basidiobolomycosis in resource-limited tropical areas where advanced mycological and molecular diagnostics are unavailable.

The present study has several limitations. First, the diagnosis was established solely on histopathological demonstration of eosinophil-rich granulomatous inflammation with broad pauciseptate hyphae and the Splendore–Hoeppli phenomenon, as microbiological cultures remained negative, precluding definitive species-level confirmation. Second, molecular diagnostic techniques (e.g., fungal PCR, DNA sequencing, or *Basidiobolus*-specific immunohistochemistry) were unavailable, limiting confirmation of the organism at the species level [19, 23]. Third, the follow-up period was relatively short for a chronic infection, and epidemiological exposure history was incomplete. Finally, the three-layered approach was applied retrospectively to a single case without a validation cohort.

### Future directions

Future investigations should focus on prospective validation of the three-layered framework in multicenter cohorts across endemic regions, with emphasis on integrating molecular diagnostics (pan-microbial PCR, metagenomic sequencing, and DNA microarrays) as a fourth confirmatory layer. Additionally, dynamic monitoring protocols should be developed to compare outcomes of medical (antifungal therapy) versus surgical (resection) treatment modalities in abdominal basidiobolomycosis.

## Data Availability

The data supporting the findings of this study are entirely included in the manuscript document.

## Abbreviations

*B. ranarum*: *Basidiobolus ranarum*
DAMPs: danger-associated molecular patterns
PAMPs: Pathogen-associated molecular patterns
